# Explainable attrition risk scoring for managerial retention decisions in human resource analytics

**DOI:** 10.3389/fdata.2025.1699561

**Published:** 2026-01-12

**Authors:** M. S. Pavithran, S. M. Vadivel

**Affiliations:** 1 School of Computer Science and Engineering, Vellore Institute of Technology, Chennai, Tamil Nadu, India; 2 Centre for Neuroinformatics, Vellore Institute of Technology, Chennai, Tamil Nadu, India; 3 VIT Business School, Vellore Institute of Technology, Chennai, Tamil Nadu, India

**Keywords:** employee attrition, predictive analytics, human resource management, risk scoring, explainable AI

## Abstract

**Introduction:**

Employee turnover remains a significant challenge for organizations as it becomes difficult for them to retain the same employees and continue with their operations efficiently. With the assistance of predictive analytics, HR managers will be able to foresee and lower the potential turnover. Conventional research has focused on the effectiveness of technical models, yet there is a lack of studies investigating the interpretability and reliability of managerial forecasts.

**Methods:**

This research used the Employee Attrition dataset and applied various pre-processing methods, including label encoding, feature scaling, and SMOTE for class balancing. Machine learning models were trained and optimized using grid search with stratified cross-validation. The best-performing model was calibrated using the sigmoid method to ensure the accuracy of the predicted probabilities. LIME enabled local interpretability, thus providing practical insights into individual employee attrition-related risks. Permutation feature importance analysis and SHAP summary plots helped in better understanding the model by showing the individual features that contributed to the attrition probability.

**Results:**

The Random Forest classifier achieved the highest AUC-ROC score of 97.37%. Risk distribution visualizations highlight employees with the highest attrition probability, and calibration is the main reason for the Brier Score reduction from 0.03873 to 0.03480.

**Discussion:**

The study concludes that by prioritizing interventions and increasing the accuracy of retention strategies, a calibrated, interpretable, and risk-stratified model can enhance HR decision-making. This framework aids HR leaders in transitioning from reactive to proactive workforce management by leveraging data-driven insights.

## 1 Introduction

The loss of employees has been a recurring and convoluted issue that companies all over the world have been facing. As companies are becoming more dependent on knowledge-based, skilled employees, the departure of the experienced ones is shaking not only the operational goals for the short term but also the strategic objectives for the long term (Manafi Varkiani et al., 2025). The rise in voluntary turnover has been influenced by the change of work culture, job discontent, and the shifting of workforce expectations (Shi et al., 2025). Employees are asking for a better work-life balance, growth in their professional career, real engagement with their work, and a competitive salary (Salem, 2025). These developments have led to the emergence of a highly mobile workforce, and employers are feeling great pressure to be proactive in retention efforts (Bloom et al., 2024).

Organizations are attrition-ridden at various hierarchical levels, which have their local and strategic impact. Besides the disrupted operations of the organization, a substantial amount of money and invisible costs are being lost. The replacement of employees brings recruitment costs, onboarding costs, training costs, and lost productivity. Besides that, high turnover rates can lower employee morale, affect teamwork, and reduce the accumulation of organizational knowledge (Gundlach, 2025). From a company's point of view, over-attrition is not only an HR problem but also a strategic risk that may lead to the loss of a company's ability to compete, grow, and innovate. To cope with these risks, enterprises have started to implement data-driven methods to foresee and alleviate employee turnover. For instance, predictive analytics offers the capability to identify employees who are most likely to quit; thus, HR personnel can systematically and timely make interventions (Wang and Huang, 2025). Nevertheless, in many cases, predictive modeling has advanced significantly, yet a majority of tools are still very technical and difficult for an average HR practitioner to understand. Even though models may provide correct forecasts, they rarely disclose the factors that cause the attrition, which diminishes the value of these models and trust in managers' decision-making (Baydili and Tasci, 2025).

Predictive models are heavily limited by one of their most important factors, their transparency, which is often missing in these models. Most of the machine learning models are so-called black boxes that just output the final results without exposing the way predictions are figured out (Mitravinda and Shetty, 2022). This becomes a problem for HR professionals, who need to provide support to leadership and, at the same time, keep fairness and accountability in employee-related actions. Even with correct models, without interpretability, it is still very challenging to implement them, especially in employee-management and retention-sensitive areas. Accurately predicted probabilities are a serious issue. In workforce planning, it is not only the question of whether an employee is going to leave, but also the degree of that likelihood that is very important for the prioritization of interventions (Konar et al., 2025). For example, HR executives might want to use the interventions for employees at the highest risk and keep the engagement activities for those at moderate risk. To do that, the predicted probabilities have to be calibrated accurately; that is, they need to be close to the actual chances taken from the observed data. Unfortunately, most of the real-world machine learning applications in the industry do not give due consideration to this point, and as a result, risk scores tend to be deceptive or unreliable.

Most of the predictive models, to begin with, portray risk in a vague or general manner, and the findings are not translated into actionable information at the individual level. Human resource managers need a technology tool that can locate the worker-level findings to facilitate targeted interventions. Sophisticated explanation techniques, such as LIME, help to close this gap by disclosing the elements that most influence the turnover of an individual worker. However, such instruments have rarely been integrated into real-world HR systems despite their potential. This research addresses these significant limitations by proposing a complete end-to-end HR-centric framework for employee attrition prediction. The suggested method mixes several machine learning models to identify employees at risk, employs probability calibration techniques to make the predictions more trustworthy, and comprises XAI tools to provide accurate individual recommendations. The emphasis is not only on the prediction precision but also on the usability and interpretability aspects; thus, the framework can be used for real-world HR decision-making.

By combining strong analytics with clear, actionable results, this study provides an actionable tool for HR leaders wishing to optimize retention initiatives. It enables and is aligned with a move from reactive to proactive human capital management, enabling organizations to construct a more stable, engaged, and future-capable workforce.

### 1.1 Research questions

- How can predictive analytics support HR managers in identifying employees at high risk of attrition?
- To what extent do calibrated risk scores enhance the reliability of attrition forecasts for informed decision-making in employee retention?
- What role do model interpretation tools play in helping HR professionals to comprehend and take action based on individual attrition risk profiles?

### 1.2 Research objectives

- To create and test a machine learning-based predictive framework that can be used by HR to identify employee attrition risk in a very accurate and reliable manner.
- To determine the worth of managerial intervention through probability calibration in maintaining the trustworthiness of predictive models employed for strategic HR moves.
- To investigate the significance of explainable AI methods in delivering to HR managers clear and actionable explanations that individual predictions of attrition have been made to them, for them to be able to implement personalized retention strategies.

The remaining parts of the paper are organized in the following manner: Section 2 goes through the related literature that is relevant to this study. Section 3 elaborates on the dataset and describes the methodology used. Section 4 presents the findings and analysis. Lastly, Section 5 wraps up the paper with a recap of the main points.

## 2 Related work

Employee turnover continues to be one of the major issues that companies worldwide are concerned about. It still heavily influences how knowledge is retained, how financially secure the company is, and its overall operational effectiveness. Hence, the use of HR analytics, especially machine learning, is turning into an efficient way to review and forecast employee attrition (Shabbir et al., 2025). Employee attrition prediction has attracted the interest of a great number of researchers, and various papers, which use machine learning, have been published, to locate the main factors of turnover and to predict the risk. The researchers have experimented with different algorithms, such as Decision Trees, Random Forests, Logistic Regression, and ensemble methods, for a variety of HR datasets. Usually, these models exhibit good predictive power, but they also differ a lot in their level of explanation, calibration, and the possibility of their use for making decisions in the HR field. Some research works use feature importance or simple explanation methods, but hardly any provide personalized insights that HR managers can execute. Table 1 displays a summary of the works that are representative of this field, indicating their approaches, results, and challenges.

**Table 1:** Summary of existing work.

**Reference**	**Methodology**	**Findings**	**Limitations**
Nagpal et al. (2024)	Supervised machine learning algorithms, including Decision Tree, KNN, Random Forest, XGBoost, SVM, and Logistic Regression, are applied to HR data to predict employee attrition.	Ensemble models like XGBoost and Random Forest demonstrate superior accuracy in forecasting employee attrition, with feature importance aiding interpretation.	Model calibration and explainability are limited, highlighting the need for further research in transparency and robustness.
Amzad Basha et al. (2024)	The study compares multiple ML models, including Logistic Regression, Random Forest, GBM, XGBoost, SVM, and KNN, on a cleansed HR dataset using accuracy, precision, recall, and F1-score metrics.	SVM and GBM models attain the best balance between accuracy and recall for the minority class, while Random Forest shows high true negative classification.	Limitations include false positive/negative trade-offs, model interpretability challenges, and practical barriers to managerial adoption of predictive analytics in HR decisions.
Raja Rajeswari et al. (2022)	This study uses KNN, Naive Bayes, Logistic Regression, and Multilayer Perceptron on the IBM HR attrition dataset using an 80–20 train-test split and feature engineering.	Logistic Regression achieves the best test accuracy of 90%, while Multilayer Perceptron performs well but may be overfitting on the small dataset.	Limitations include potential overfitting, limited dataset size, lack of temporal data, and practical challenges in managerial adoption of predictive systems.
Angulakshmi et al. (2024)	Decision Trees and Random Forests are applied to cleansed HR datasets to forecast attrition, satisfaction, and workforce needs using machine learning metrics.	Machine learning models reveal that higher education, seniority, and better pay reduce attrition while enhancing satisfaction; predictive analytics supports strategic workforce planning.	Challenges include data privacy, ethical biases, model integration, and explainability, indicating a need for improved calibration and XAI in predictive HR analytics.
Juhi Padmaja et al. (2022)	The study uses IBM HR Analytics data with imbalanced classes, applying Random Oversampling, Random Undersampling, and SMOTE sampling techniques alongside classifiers like Logistic Regression, KNN, Decision Tree, Random Forest, and AdaBoost.	AdaBoost with SMOTE sampling achieves the highest AUC score, outperforming other classifiers across different sampling methods in predicting employee attrition.	Limitations include imbalanced data challenges, model calibration needs, and managerial adoption issues related to trust and interpretability of ML predictions.
Habous et al. (2021)	The paper applies supervised machine learning classifiers, including Gaussian Naive Bayes, Bernoulli Naive Bayes, Decision Tree, Logistic Regression, and Random Forest, on IBM HR attrition data with detailed preprocessing and feature selection.	Gaussian Naive Bayes achieved the best recall of 70.76% minimizing false negatives, while Random Forest had high accuracy but lower recall for attrition prediction.	The study notes challenges with class imbalance, need for model calibration, explainability of predictions, and considerations for managerial adoption of predictive analytics tools.
Cherian et al. (2025)	The study uses IBM HR Analytics data with J48 decision tree for feature selection and evaluates six tree-based classifiers, including Random Forest, Hoeffding Tree, Rep Tree, and Logistic Model Tree.	Random Forest achieves the highest test accuracy of 87.07%, outperforming other tree classifiers by effectively capturing complex attrition patterns.	Limitations include class imbalance, false positives and negatives trade-offs, and challenges in managerial adoption and model explainability.
Jin et al. (2024)	The study applies logistic regression, random forest, decision tree, K-nearest neighbors, bagging, stacking, and rotation forest on the IBM HR dataset with hyperparameter tuning to predict employee attrition.	Random forest achieves the highest accuracy of 95% with key attrition factors identified as overtime, stock option level, and job satisfaction; ensemble methods like bagging and stacking also show strong performance.	Limitations include model complexity, need for further calibration, explainability challenges, and practical barriers to managerial adoption and integration of predictive analytics.
Aggarwal and Goyal (2023)	The study develops a Random Forest-based predictive attrition scoring model for top performers in financial services using a single employee view from over 8,000 employees, incorporating HR, psychological, and performance variables.	Model prediction accuracy ranges from 61% to 67% over a three-month window, with LIME explanations identifying key attrition drivers like burnout, incentives, and supervisor support for tailored retention strategies.	Limitations include moderate prediction accuracy, challenges in model calibration, explainability, and practical managerial adoption for effective retention interventions.
Sivakarthi et al. (2024)	This study employs logistic regression along with decision trees and random forests on comprehensive MNC employee data after extensive preprocessing and feature engineering to predict attrition.	The logistic regression-based model achieves a reliable prediction accuracy of 64.6% and identifies key attrition factors such as department, education, job role, and age, enabling targeted retention strategies.	Limitations include moderate accuracy, the necessity for model recalibration, explainability challenges, and managerial adoption complexities, highlighting areas for future improvement.
Liang and Kang (2024)	The paper proposes integrating a Test-Time Training layer with baseline classifiers like Random Forest, SVM, and Logistic Regression, utilizing the IBM HR dataset for improved prediction adaptability and accuracy.	Integrating the TTT layer significantly enhances accuracy, precision, and recall metrics across all base classifiers, demonstrating better performance on unseen test data.	Limitations include variability of TTT effectiveness with dataset characteristics, binary classification challenges, and the need for further work on model explainability, calibration, and managerial adoption.
Vij et al. (2023)	This paper uses a multidisciplinary approach combining data collection from HR records and surveys, exploratory data analysis, hierarchical clustering, and machine learning models, including logistic regression, decision trees, Random Forest, Gradient Boosting, and deep learning for predictive modeling of female employee attrition in private education colleges.	Work-life balance, career advancement opportunities, and organizational culture significantly influence attrition, with predictive models such as logistic regression and decision trees effectively forecasting attrition risks and enabling proactive retention strategies.	Limitations include model complexity, need for ongoing model calibration, explainability challenges, and barriers to managerial adoption of predictive analytics findings for attrition reduction.
Muralidharan et al. (2024)	This empirical study utilizes the Random Forest algorithm on the Kaggle HR dataset with preprocessing, feature selection via correlation matrices, and fairness evaluation through disparate impact metrics.	The model demonstrates effective prediction of employee attrition while maintaining fairness across sex and race, highlighting key factors like performance, satisfaction, and absenteeism.	Challenges include balancing efficiency with ethical fairness, ongoing bias detection, transparency in algorithmic decisions, and practical barriers to managerial adoption of ethical AI in HR.
Kambhampati and Rao (2024)	This systematic review integrates advanced machine learning techniques, including CatBoost, Random Forest, deep learning, and feature selection methods, applied to diverse HR datasets supplemented by employee surveys and exit interviews.	The review highlights the superior predictive performance of ensemble and deep learning models, the efficacy of advanced feature selection, and emphasizes the importance of ethical, privacy-aware frameworks in HR attrition analytics.	The review notes gaps in transparency, comparability, and explainability of models, and underscores practical challenges in ethical data usage and managerial adoption of AI-driven HR analytics.
Pratibha and Hegde (2022)	Kernel Principal Component Analysis was used for dimensionality reduction, Adaptive K-means for clustering, and Logistic Regression for classification to predict employee attrition.	The KPCA-Adaptive K-means-Logistic Regression model outperforms Naive Bayes and standard Logistic Regression with 96.9% accuracy, showing improved clustering and classification results.	The paper notes limitations in interpretability, model explainability, and calibration, along with challenges in managerial adoption of the predictive analytics system.
Manikandan et al. (2023)	The study proposes a novel Hybrid Learning based Employee Attrition Prediction model combining deep learning techniques such as ANN and RNN with extensive preprocessing, normalization, regularization, and k-fold cross-validation on HR data.	HLEAP outperforms traditional ANN and RNN models with accuracy up to 96.84%, recall around 95.93%, and F1-score near 93.84%, demonstrating robust early attrition prediction.	Limitations include model complexity, the requirement of periodic tuning for new data, challenges in explainability and calibration, and managerial adoption hurdles for practical deployment.
Mehta and Modi (2021)	This study employs tree-based ensemble methods, specifically Random Forest and Gradient Boosting, on the IBM HR Analytics dataset, incorporating thorough preprocessing, feature selection via correlation analysis, and model training with scikit-learn.	The Gradient Boosting classifier outperforms Random Forest, achieving around 95% accuracy while balancing precision and recall better on the imbalanced attrition dataset.	Limitations include dependency on medium-sized datasets, the need for additional feature inclusion, computational complexity, and challenges in explainability and managerial adoption of predictive models.
Mitravinda and Shetty (2022)	The study employs multiple machine learning classifiers, including Logistic Regression, Decision Tree, Random Forest, KNN, AdaBoost, XGBoost, and Gradient Boosting on the IBM HR dataset with 7-fold cross-validation and SHAP values for feature importance.	XGBoost provides the best predictive performance of accuracy 87.3% and SHAP analysis identifies OverTime, MonthlyIncome, StockOptionLevel, and Age as key drivers of attrition.	Limitations include model complexity, need for continuous calibration, challenges in explainability, and practical issues around managerial adoption for implementing retention recommendations.
Singh Satia et al. (2019)	The study uses a Decision Tree with AdaBoost on a dataset collected via survey from 498 teachers in Delhi, analyzing 10 features including age, gender, qualification, salary, attendance, and work satisfaction.	The AdaBoost-enhanced Decision Tree achieves 90% accuracy in predicting teacher attrition; key factors negatively correlated with attrition include age, experience, salary, attendance, and work satisfaction.	The study is limited by dataset size and region, model complexity, the need for further feature inclusion, and challenges in managerial adoption of predictive interventions.
Younis et al. (2024)	This study combines traditional HR metrics with social network analysis and graph theory, computing network centralities from relational network data of 169 employees, and uses stepwise regression to predict attrition intent among future leaders and subject matter experts.	Network-enhanced models improve the prediction accuracy of employee attrition, revealing that subject matter experts with high centrality have higher attrition intent, while future leaders' advice-seeking behavior is negatively correlated with attrition; perceived investment in employee development mediates these effects.	Limitations include the study's scope limited to one organization, reliance on cross-sectional data, complexity of social network measures, and challenges in managerial adoption of network-based predictive analytics.
Govindarajan et al. (2025)	Implemented a hybrid machine learning approach combining Random Forest, SVM, Decision Tree, and Gradient Boosting algorithms, applied SMOTE to balance the imbalanced dataset, and used an 85:15 train-test split for evaluation.	The hybrid model achieved the highest accuracy of 95%, outperforming individual models, demonstrating that combining multiple algorithms improves prediction effectiveness for employee attrition.	The use of SMOTE affects model probability calibration, the hybrid model's complexity reduces interpretability and explainability, and the lack of direct integration with HR management limits practical adoption by managers.
Ding et al. (2025)	Analyzed 19,186 Glassdoor employee reviews using Structural Topic Modeling to extract themes and Support Vector Machine to validate topic impact on satisfaction.	Positive factors included good supervision and career growth; negatives were cultural challenges, unfair treatment, and inflexible policies.	Model calibration is complex due to topic modeling assumptions; explainability is limited by probabilistic topics and SVM opacity.
Park et al. (2024)	Analyzed turnover intention of new college graduates with logistic regression, K-nearest neighbor, and extreme gradient boosting (XGB) classifiers; model training used a 70:30 train-test split with 4-fold cross-validation.	Job security satisfaction was the strongest predictor of turnover intention, followed by organizational satisfaction and job-major fit; the XGB model achieved the highest accuracy of 78.5%.	Model calibration may be affected by data imbalance; XGB provides feature importance but lacks a clear causal direction, limiting explainability.
Bhuria et al. (2025)	The study used a Voting Classifier ensemble combining Random Forest, Decision Tree, Support Vector Classifier, K-Nearest Neighbors, and XGBoost on a balanced dataset created using SMOTE, and extensive exploratory data analyses and outlier removal with IQR were performed for data preparation.	The ensemble Voting Classifier with SMOTE achieved a superior accuracy of 90%, precision, recall, and F1-score of 0.90, outperforming individual classifiers and effectively predicting bank customer churn.	Calibration is affected by synthetic data balancing. Explainability is limited by the complexity of ensemble models, despite the use of *post-hoc* methods like SHAP.
Talebi et al. (2025)	The study proposes a hybrid model combining Genetic Algorithms for optimal feature selection with LightGBM for employee turnover classification. Genetic Algorithms used a population-based search to select informative features, while LightGBM applied leaf-wise gradient boosting for classification with hyperparameters tuned by grid search.	The hybrid model achieved an AUC of 0.94 and an F1-score of 0.87 on the public dataset and performed well on the proprietary dataset, outperforming traditional models such as Random Forest, Logistic Regression, and standalone LightGBM.	Although the hybrid model improves interpretability through feature selection, it remains complex, with limited transparency. Managerial adoption may be challenging due to the technical nature of the approach and the need for HR analytic expertise.
Nayem and Uddin (2024)	The study collected data from 1,109 employees of Bangladeshi for-profit organizations, covering physical, social, and economic factors affecting performance. Seven machine learning models were trained on this data after preprocessing with feature scaling and encoding.	Random Forest had the highest accuracy of 98.2%, followed by Gradient Boosting and K-NN. Important factors positively correlated with performance included training facilities, age, salary increment, work experience, promotions, and work environment conditions.	Calibration may be affected by class imbalance. Explainability varies as tree-based models offer some interpretability, but other models like SVM and Naive Bayes are less transparent.
Biswas et al. (2023)	The study collected survey data from 597 IT employees and used an ensemble learning model to predict the intention to quit. Data preprocessing included feature scaling and sampling to balance classes. The study also applied SOR theory to understand behavioral patterns. Information gain and correlation analyses were used to identify key predictors.	The ensemble model achieved 94.7% accuracy and outperformed individual models. Key predictors for IQ included activities on professional networking sites, job involvement, and updates on job portals. The study found that social media use and job dissatisfaction are strong signals of intention to quit, as framed by the SOR theory.	The model is limited to IT professionals and doesn't include demographic variables. Managerial adoption requires adapting insights into actionable HR policies and expanding model scope beyond the current sample.

Yahia et al. (2021) used a mixed method combining employee survey, feature selection algorithms, machine learning, deep learning, and ensemble learning models to predict employee attrition, finding that a deep data-driven approach focusing on key features improves prediction accuracy and reveals business travel as a top retention motivator beyond traditional rewards. Uliyan et al. (2021) applied deep learning techniques using bidirectional LSTM and conditional random fields to university student datasets to predict student retention and dropout with high accuracy, demonstrating the effective early identification of at-risk students to support intervention planning. Mortezapour Shiri et al. (2025) proposed an ensemble deep learning model based on a Bidirectional Temporal Convolutional Network with GAN-based data augmentation to predict employee attrition, achieving high accuracy on IBM and Kaggle datasets while identifying key attrition features using XAI.

This review showcases how the literature has evolved, moving from simple classifiers to hybrid and neural architectures. They mirror an increasing focus on predictive HR analytics, but disclose that the openness of the model, calibration of probability, and usability for management remain significant gaps. The majority of the research works favor accuracy at the cost of reliability, and very few have adopted explainable AI techniques that facilitate personalized retention strategies. Moreover, challenges such as class imbalance, ethical fairness, and integration with HR workflow remain underexplored. These limitations underscore the need for a comprehensive framework that combines high-performance prediction with calibrated risk scoring and actionable interpretability, which was the focus of this study.

### 2.1 Research gaps

While there is a lot of talk about using data to inform human resource decisions, the methods used to predict employee attrition in general fail to address three key areas that are of interest to human resource management:

- Too much focus on accuracy and not enough on reliability: Many of the existing tools produce binary predictions (stay/leave) without showing the model's level of confidence, thereby restricting its use for nuanced HR planning. Calibrating probability outputs so that they accurately represent real-world risks is an area that has not been sufficiently explored.
- Lack of sufficient transparency in models used for prediction: The so-called black-box algorithms are getting increasingly popular; however, most HR departments do not have the technical capability to understand these models. Research does not engage interpretability frameworks such as LIME and SHAP, which are necessary for managerial trust and adoption.
- Individualized risk communication being ignored: Although attrition prediction has been researched on a general level, very little has been done regarding the presentation of employee-specific risk scores that would facilitate proactive HR intervention.

The present research project is designed to address these issues by setting up an interpretable, reliable, and HR-centric attrition prediction system that is geared toward strategic decision-making and individualized retention planning.

### 2.2 Contributions of the research

This research presents a novel human resource-centric machine learning framework, integrating probability calibration with multilevel explainability instruments to facilitate strategic employee retention. While earlier studies have focused on predictive modeling, this research differentiates itself by the following innovations:

- Dual-layer interpretability: The presented model combines global interpretability based on SHAP summary plots and permutation feature importance with local employee-specific explanations via LIME. This allows not only strategic workforce insights but also personalized retention actions—an uncommon combination in HR analytics.
- Calibrated risk scoring for managerial triage: Through the application of sigmoid calibration, the model converts its raw predictions into reliable probability estimates. Hence, HR professionals can decide on interventions based on the actual attrition risk rather than on a binary classification.
- Permutation-based feature ranking for HR transparency: By engaging permutation importance, the study grants easy-to-understand, model-agnostic insights into the most influential factors for attrition, thus improving managerial trust and interpretability.
- SHAP-based global insights for strategic planning: SHAP values demonstrate the features that lead to attrition not only for the current workforce but also, thus, aiding long-term organizational retention planning.
- End-to-end deployment-ready pipeline: This research provides a thoroughly reproducible workflow, including preprocessing, SMOTE balancing, calibration, and explainability steps, designed for real-world HR practice rather than a mere academic experiment.
- Managerial usability as a design goal: In contrast to previous works that concentrated on technical performance, this framework is specifically intended to serve non-technical HR stakeholders, with visualizations and explanations that facilitate ethical, transparent, and actionable decision-making.

## 3 Methodology

**Figure 1:**
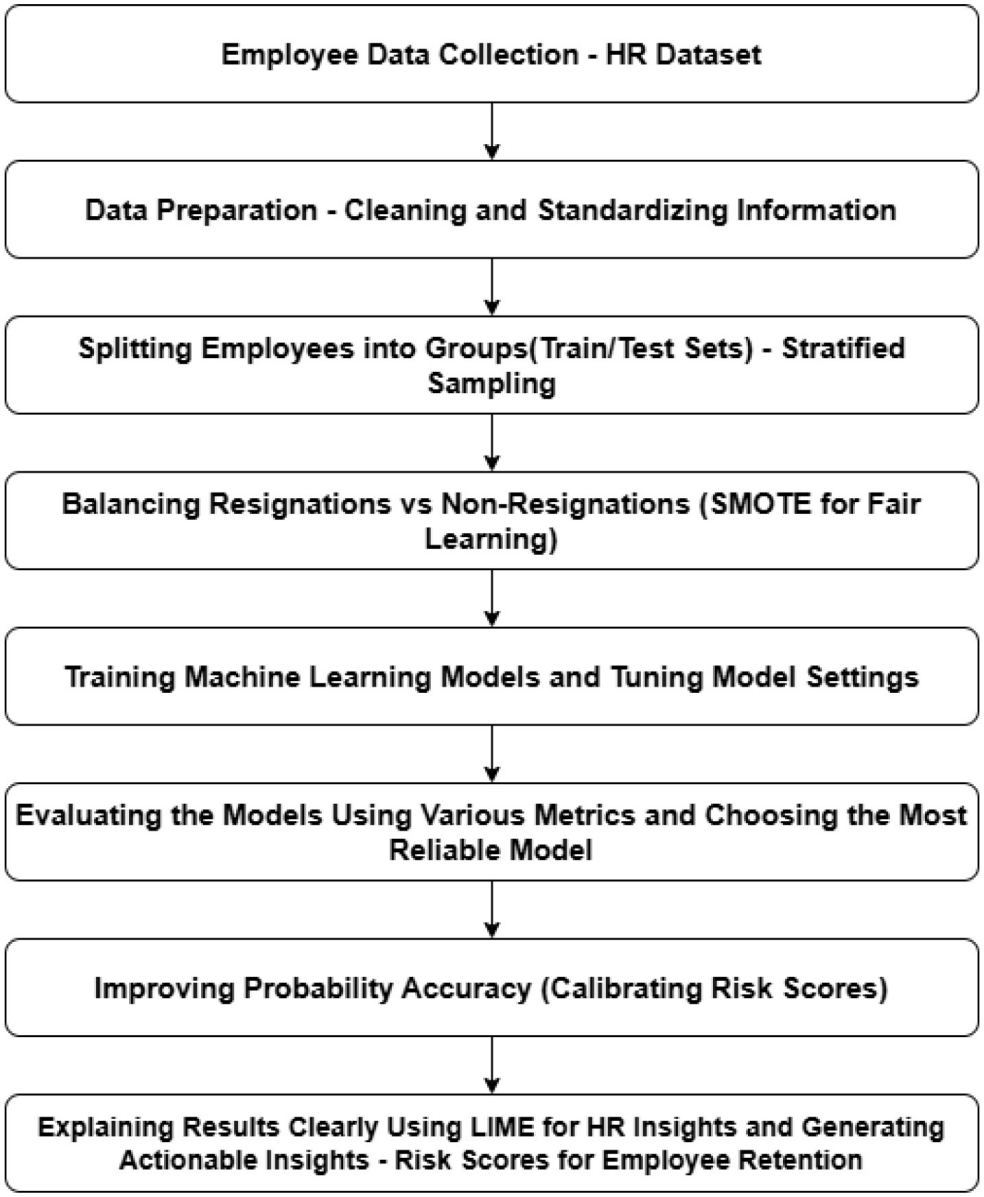
Flowchart of the proposed methodology.

Emphasizing the predictive modeling of employee attrition utilizing interpretable and calibrated machine learning approaches, this study employed an organized methodology designed to meet the objectives of HR professionals. In order to ensure uniformity, the dataset was initially pre-processed by means of feature scaling and label encoding. To rectify the class imbalance, SMOTE was employed for oversampling. Different machine-learning models were trained and tuned via a grid search with stratified cross-validation. The models were evaluated using different metrics with an emphasis on the identification of employees at high risk. To further calibrate the best-performing model and thus increase probability accuracy as reflected by the Brier score, the sigmoid method was employed. In the conclusion, HR-friendly explanations for individual predictions were made possible through the use of LIME, thus facilitating actionable insights for retention planning. The complete workflow of the proposed HR attrition prediction framework, which comprises data pre-processing, model training, calibration, and explainability, is represented in Figure 1.

### 3.1 Data collection

**Figure 2:**
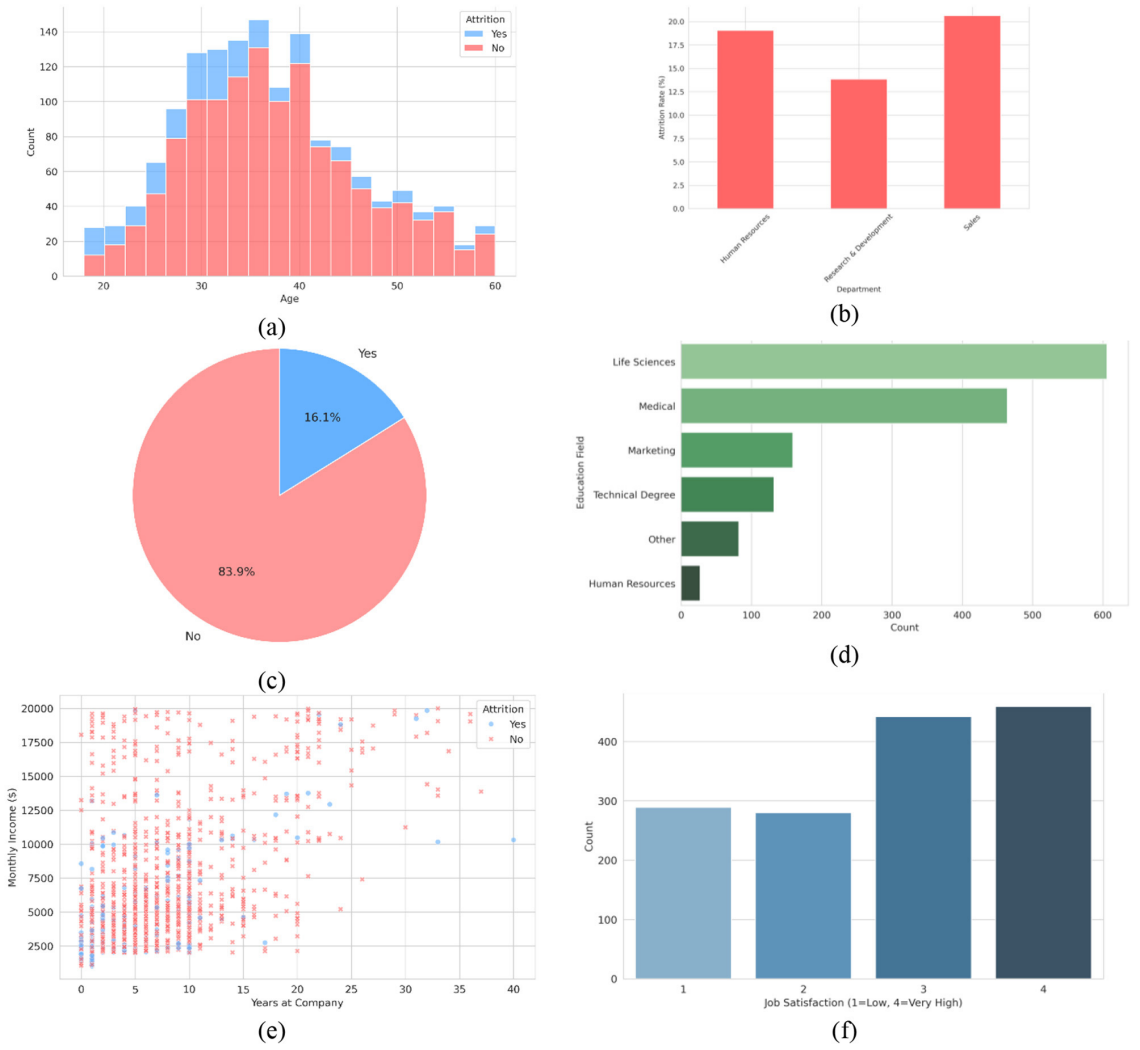
Visualizations of employee data: **(a)** age distribution by attrition, **(b)** attrition rate by department, **(c)** employee attrition distribution, **(d)** employee distribution by education field, **(e)** monthly income vs. years at company by attrition, and **(f)** job satisfaction distribution.

An Employee Attrition dataset from Kaggle was utilized to conduct a study on employee turnover and organizational behavior. The dataset includes 1,470 employee records, where each record corresponds to a single employee and consists of 13 main attributes. These attributes describe demographic, professional, and workplace satisfaction metrics; thus, the dataset is appropriate for investigating the causes of employee attrition. The dataset was instrumental in building a predictive model and conducting exploratory data analysis. Table 2 presents a summary of the main attributes of the dataset. The Dataset visualization is presented in Figure 2.

**Table 2:** Summary of key attributes.

**Attribute**	**Type**	**Description**	**Range/values**
Age	Continuous	Employee's age in years	18–60 years
Attrition	Categorical	Indicates if the employee left the company	Yes, No
Department	Categorical	Employee's department	Sales, Research and Development, Human Resources
DistanceFromHome	Continuous	Distance from the employee's home to the workplace in miles	1–29 miles
Education	Ordinal	Education level of the employee	1 (Below College), 2 (College), 3 (Bachelor's), 4 (Master's), 5 (Doctorate)
EducationField	Categorical	Field of study of the employee	Life Sciences, Medical, Marketing, Technical Degree, Human Resources, Other
EnvironmentSatisfaction	Ordinal	Satisfaction with the work environment	1 (Low), 2 (Medium), 3 (High), 4 (Very High)
JobSatisfaction	Ordinal	Satisfaction with the job	1 (Low), 2 (Medium), 3 (High), 4 (Very High)
MaritalStatus	Categorical	Employee's marital status	Single, Married, Divorced
MonthlyIncome	Continuous	Employee's monthly income in dollars	$1,000–$19,999
NumCompaniesWorked	Continuous	Number of companies the employee worked for previously	0–9 companies
WorkLifeBalance	Ordinal	Employee's work-life balance rating	1 (Bad), 2 (Good), 3 (Better), 4 (Best)
YearsAtCompany	Continuous	Number of years the employee has been with IBM	0–40 years

### 3.2 Data preprocessing

Before developing the model, the dataset was subjected to several preprocessing steps to make it ready for analysis. Label encoding was used to convert categorical data into a numerical format. The numerical features were normalized so that each variable could make an equal contribution to the model without being influenced by the magnitude. This step is very important to machine learning algorithms stability and performance. A stratified method was used to divide the dataset into training and testing sets; thus, the ratio of employees who left to those who remained was kept unchanged. This stratification was necessary due to the imbalance of the attrition variable. Training was done with 80% of the data, and testing was done with 20%. Such a division is a guarantee that models will be tested on data that is new to them, and thus an objective evaluation of the models' prediction ability will be possible.

### 3.3 Handling class imbalance

Attrition datasets frequently reveal that the classes are imbalanced, such that the number of employees who leave is considerably less than the number of employees who stay. The issue was fixed by applying a Synthetic Minority Oversampling Technique. SMOTE creates new examples of the target classes by combining the existing samples. In this way, the models get better at detecting actual cases of attrition as they are not allowed to become biased toward the dominant class.

### 3.4 Model selection and training

K-Nearest Neighbors, Decision Tree, Random Forest, Support Vector Machine, AdaBoost, Gradient Boosting, XGBoost, LightGBM, and Multi-layer Perceptron were among the nine different classification techniques considered for comparison. Predictive power and interpretability were balanced in the selection of these models.

### 3.5 Cross-validation and hyperparameter optimization

The efficiency of each model is influenced by particular settings or hyperparameters. In order to figure out the best configurations, a Grid Search with 5-fold Stratified Cross-Validation was performed. This method goes through parameter combinations in a very detailed way to find the one that leads to the highest performance. Cross-validation was used to make sure that the models were not overfit to a certain part of the data.

### 3.6 Evaluation metrics

The models were evaluated using several metrics relevant to HR decision-making.

- Accuracy: The overall correctness of the predictions.
- Recall: The proportion of the actual attrition cases correctly identified.
- Precision: Accuracy of predicted attrition cases.
- F1-Score: Harmonic mean of precision and recall.
- AUC-ROC Score: Measures the model's ability to distinguish between classes.

Confusion matrices and ROC curves were plotted to visualize performance trade-offs.

### 3.7 Model calibration and attrition risk scoring

Calibration was done using the sigmoid method on the best-performing model to make the prediction probabilities more understandable. This operation modifies the simple probability scores so that they are more in line with the actually observed attrition rates. The quality of the calibration was checked with the Brier Score, a measure that evaluates the correctness of probabilistic predictions. Individual-level attrition risk scores were created for all employees after the calibration. These scores were probabilities that managers could use to decide the most efficient retention methods. A risk distribution histogram and a top-10 high-risk employee list were generated. The outputs are turning into immediate proactive HR actions such as retention bonuses, employee engagement, and mentoring.

### 3.8 Explainable AI

LIME served as a bridge between technical models and HR decision-making. LIME determines the major influences for each employee's risk score and generates easily understandable explanations for each prediction. Giving personalized insights to managers on the risk of attrition, thus, increases their trust in the system and makes them more likely to take action. In order to guarantee the transparency and interpretability of the model predictions, SHAP summary plots were created to illustrate the extent and the direction of each feature's influence on employee attrition. Moreover, permutation feature importance was obtained by altering feature values at random in order to evaluate the reduction in model performance, thus confirming feature relevance from a model-agnostic perspective. By means of these interpretability methods, the understanding of the model's predictive behavior is enhanced both from a global and a local perspective.

## 4 Results and analysis

An extensive evaluation scheme was established to figure out the efficiency of different machine learning models in predicting employee attrition. As a result, in order to provide the best possible assistance to management in their decision-making by identifying the most reliable and understandable predictive method, the findings are disclosed with an HR-focused interpretation.

### 4.1 Experimental setup

The experiment was conducted in Python 3.12.11 on Google Colab with a Tesla T4 GPU. We tested nine machine learning classifiers, and the hyperparameter tuning for each model was done using a grid search and cross-validation over a predefined search space to find the best configuration. The data was prepared by standard scaling and encoding, where necessary. To maintain the class distributions, stratified 5-fold cross-validation was used for a reliable performance evaluation.

### 4.2 Performance evaluation

Machine learning classifiers were trained on SMOTE-balanced data, and the results were judged through five-fold stratified cross-validation. To measure the effectiveness, five crucial metrics relevant to HR stakeholders were employed. Table 3 contains the percentage figures of the metrics for attrition prediction for each classifier model.

**Table 3:** Performance metrics of machine learning models.

**Model**	**Accuracy**	**Precision**	**Recall**	**F1 score**	**AUC-ROC**
Random Forest	96.3265	92.5581	83.9662	88.0531	97.3763
KNN	92.3129	70.8054	89.0295	78.8785	96.5134
LightGBM	96.3946	93.3962	83.5443	88.1960	95.1715
XGBoost	96.5306	93.8679	83.9662	88.6414	94.4470
Gradient Boosting	96.4626	93.0233	84.3882	88.4956	93.6226
MLP	95.3061	85.8974	84.8101	85.3503	93.3454
SVM	92.1769	72.5926	82.7004	77.3176	91.0332
Decision Tree	86.8027	57.6512	68.3544	62.5483	87.9386
AdaBoost	80.7483	41.7857	49.3671	45.2611	76.6832

XGBoost was the top model to achieve a very high sensitivity and specificity, thereby giving us a 96.53% accuracy and an AUC-ROC of 94.44%. In addition, precision and recall are good in nature; thus, the algorithm is extremely reliable in detecting high-risk cases with a very small number of false positives and false negatives. The results revealed that ensemble methods with better predictive power, like XGBoost, LightGBM, and Random Forest, were able to produce consistently higher AUC-ROC values. Along with XGBoost, Random Forest, LightGBM, and Gradient Boosting also displayed strong performance and high accuracy. These ensemble models would be the most suitable for HR departments that are looking for reliable and scalable ways to identify attrition risks early on. The competitive performance of neural network-based models, such as MLP, shows that they have the capacity to capture complex relationships in employee data. The hyperparameter search space and optimal configurations for each model are presented in Table 4, which is in line with standard grid search optimization protocols for reproducibility.

**Table 4:** Hyperparameter search space and optimal configurations for each model.

**Model**	**Hyperparameters search space**	**Optimal configurations**
K-Nearest Neighbors	n_neighbors: [3, 5, 7], weights: [‘uniform', ‘distance']	n_neighbors: 7, weights: distance
Decision Tree	max_depth: [3, 5, 10, None], min_samples_split: [2, 5, 10]	max_depth: 10, min_samples_split: 10
Random Forest	n_estimators: [100, 200], max_depth: [5, 10, None], min_samples_split: [2, 5]	n_estimators: 200, max_depth: None, min_samples_split: 2
Support Vector Machine	C: [0.1, 1, 10], kernel: [‘linear', ‘rbf'], gamma: [‘scale', ‘auto']	C: 10, kernel: rbf, gamma: scale
Gradient Boosting	n_estimators: [100, 200], learning_rate: [0.01, 0.1], max_depth: [3, 5]	n_estimators: 100, learning_rate: 0.1, max_depth: 5
LightGBM	num_leaves: [31, 50], learning_rate: [0.01, 0.1], n_estimators: [100, 200]	num_leaves: 50, learning_rate: 0.1, n_estimators: 200
AdaBoost	n_estimators: [50, 100], learning_rate: [0.01, 0.1, 1]	n_estimators: 100, learning_rate: 1
XGBoost	n_estimators: [100, 200], learning_rate: [0.01, 0.1], max_depth: [3, 6]	n_estimators: 200, learning_rate: 0.1, max_depth: 6
MLP	hidden_layer_sizes: [(50,), (100,), (100, 50)], activation: [‘relu', ‘tanh'], solver: [‘adam'], alpha: [0.0001, 0.001]	hidden_layer_sizes: (100, 50), activation: relu, solver: adam, alpha: 0.001

**Figure 3:**
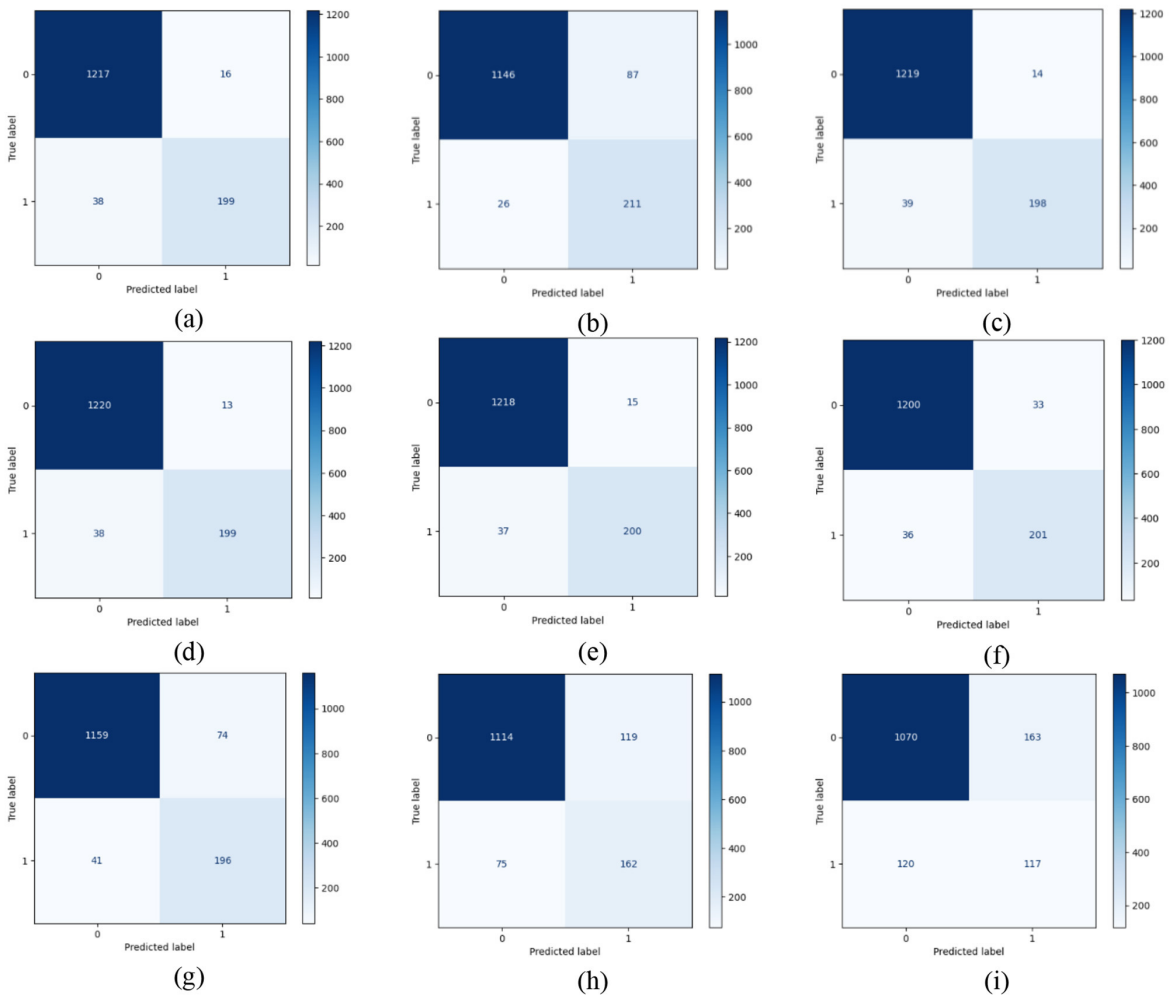
Confusion matrix of machine learning models for employee attrition prediction: **(a)** Random Forest, **(b)** KNN, **(c)** LightGBM, **(d)** XGBoost, **(e)** gradient boosting, **(f)** MLP, **(g)** SVM, **(h)** Decision Tree, and **(i)** AdaBoost.

The confusion matrix for each model was graphically represented to interpret its predictive behavior. This gave a chance to HR professionals to comprehend the compromises between false positives and false negatives. The confusion matrices of the machine learning models employed for employee attrition prediction are shown in Figure 3.

**Figure 4:**
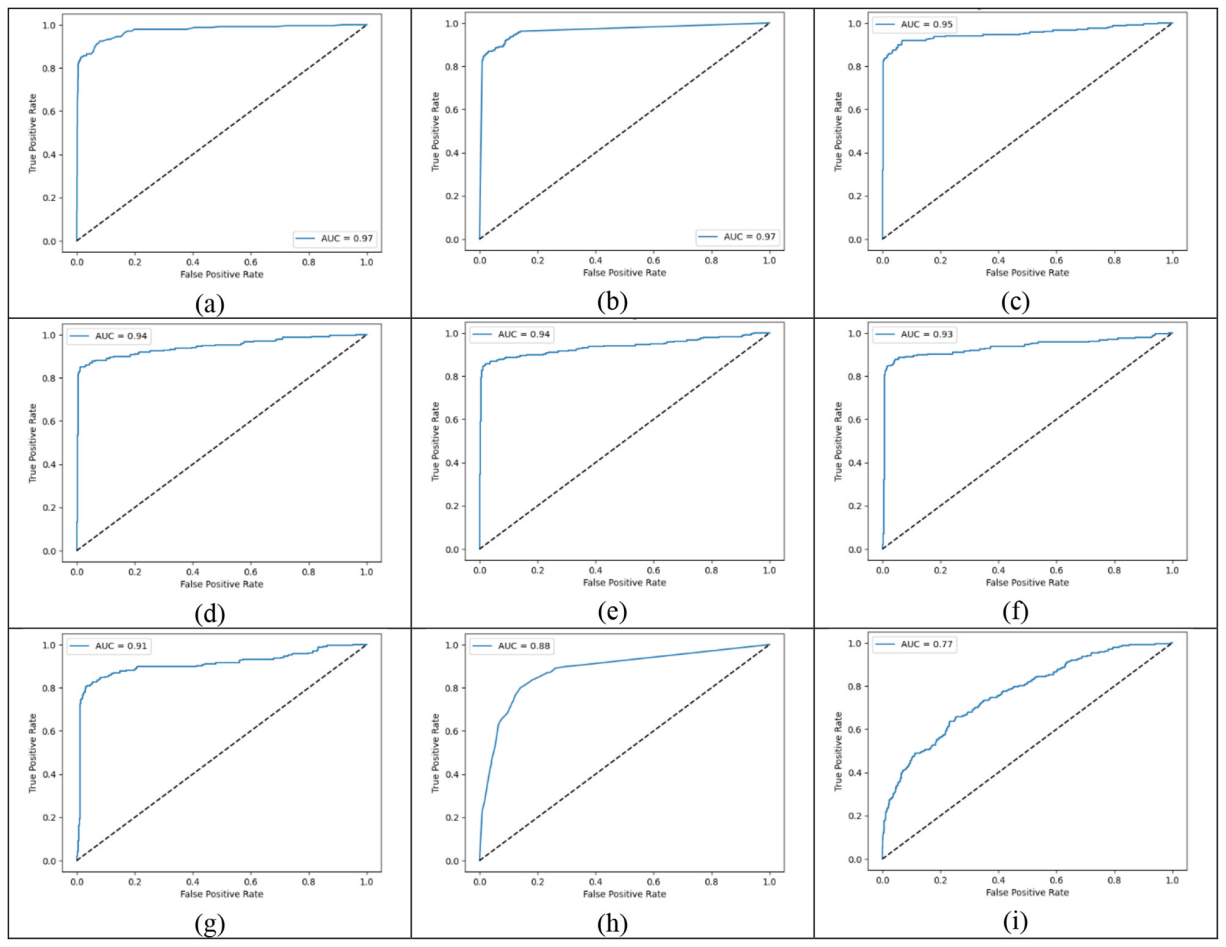
ROC curves of machine learning models for employee attraction prediction: **(a)** Random Forest, **(b)** KNN, **(c)** LightGBM, **(d)** XGBoost, **(e)** gradient boosting, **(f)** MLP, **(g)** SVM, **(h)** Decision Tree, and **(i)** AdaBoost.

The ROC curves were clear evidence that the gradient boosting models outperformed others with significantly larger AUC areas. As an example, the XGBoost ROC curve was closest to the top-left corner of the graph, thus indicating that the model was very effective in distinguishing between classes. Figure 4 shows the employee attrition prediction machine learning models' ROC curves.

**Figure 5:**
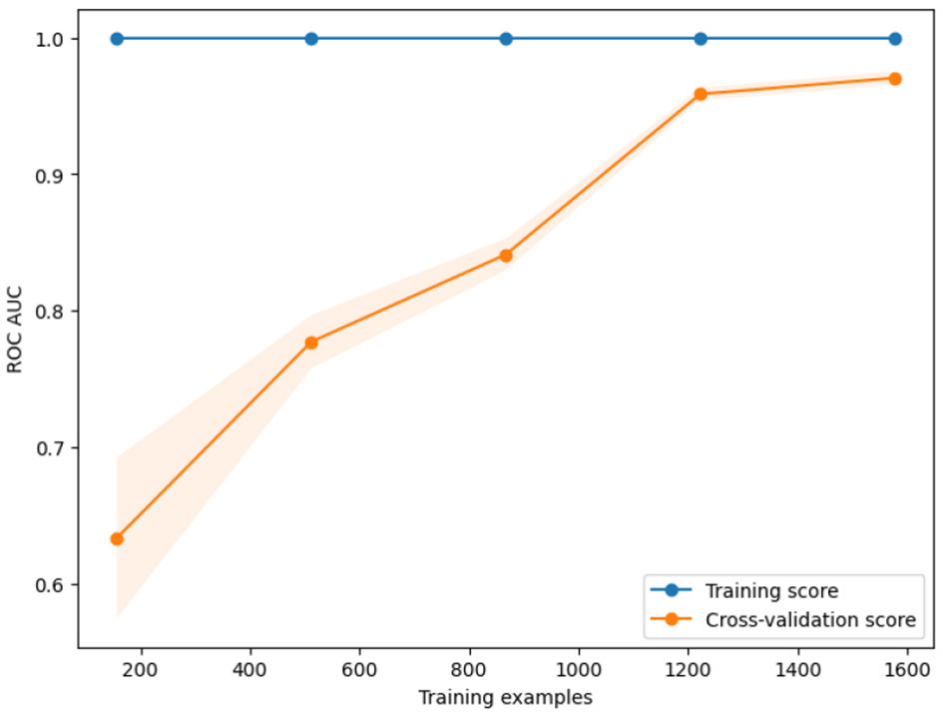
Learning curve of Random Forest model based on ROC AUC performance.

Figure 5 presents the learning dynamics of the Random Forest classifier, the ROC AUC being the performance metric. The number of training examples is on the x-axis, going from 200 to 1600, while the y-axis shows the corresponding ROC AUC scores. The shaded region around the cross-validation curve indicates the variability or the confidence intervals from the different folds. The increasing distance between the training and validation scores at lower sample sizes illustrates the model's initial overfitting, which fades as the amount of data grows.

### 4.3 Paired t-test

We carried out a paired t-test on the 5-fold ROC AUC scores of the two methods obtained on the SMOTE-resampled training set to check if the differences in performance between Random Forest and XGBoost are statistically significant. Table 5 shows the ROC AUC scores for each fold as well as the average and the standard deviation for each model.

**Table 5:** 5-fold cross-validated ROC AUC scores for Random Forest and XGBoost models.

**Model**	**Fold 1**	**Fold 2**	**Fold 3**	**Fold 4**	**Fold 5**
Random Forest	97.85	97.05	96.83	96.72	96.78
XGBoost	95.32	94.54	95.06	95.76	95.07

The null hypothesis posited that there were no differences in average ROC AUC between models, whereas the alternative hypothesis suggested that Random Forest has a higher average ROC AUC. The obtained test results included a t-statistic of 6.49 and a *p*-value of 0.0029, which meets the standard for statistical significance (*p* < 0.05). Such a small *p*-value indicates that the performance advantage of the Random Forest over the different folds is very consistent and of such a magnitude that it is highly unlikely that the observed difference is a result of random variation. Consequently, the decision was made to reject the null hypothesis in favor of the alternative hypothesis, and hence, the Random Forest was the one to be further examined in the calibration analysis.

### 4.4 Calibration and risk scoring

Random Forest was chosen for calibration in this research because of its strong overall predictive performance and practical relevance from an HR managerial standpoint. Among all models evaluated, Random Forest achieved the highest AUC-ROC score. This indicates that Random Forest consistently identifies both positive and negative cases of attrition with high reliability and minimal bias, making it a trustworthy model for critical decisions, such as employee retention planning. AUC-ROC is preferred over accuracy because it offers a balanced, threshold-independent, and interpretable view of model performance, which is especially crucial in employee attrition prediction, where both correct identification and misclassification costs have real business consequences.

From a calibration perspective, Random Forest is a very strong ensemble learning technique, but, still, as with most tree-based methods, it normally yields ill-calibrated probability estimates, and its predicted probabilities can be either overconfident or underconfident. The problem is that in HR settings, where the risk probabilities need to be very accurate, it can become an issue. For instance, if the risk of an employee leaving is at 76%, rather than just putting the label “high risk”, the HR managers would be able to come up with the interventions more logically and by using the resources most efficiently, and even by crafting the retention strategies based on how much the risk is.

In order to make the random forest better, probability calibration was used along with CalibratedClassifierCV. The model's predicted probabilities after calibration were more consistent with the real observed frequencies. This was also the case of the Brier Score, which got better after the adjustment and also the inspection of the calibration curves, which showed closer proximity to the ideal diagonal line. The Brier Score, an indicator of probability correctness, got better after the adjustment, as the score was reduced from 0.03873 (uncalibrated) to 0.03480 (calibrated), which represents a highly valuable factor for HR managers who are dependent on risk percentages when making choices regarding intervention.

**Figure 6:**
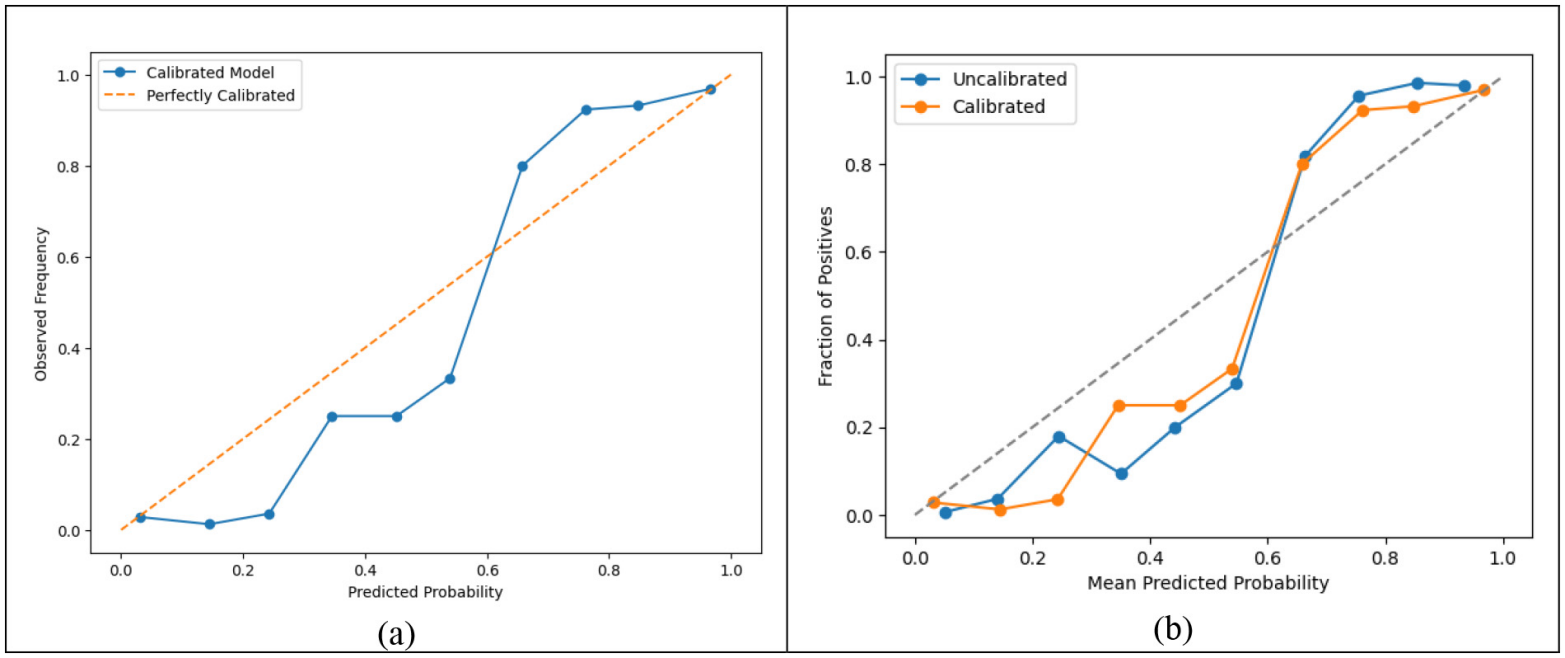
Calibration curves. **(a)** Calibration curve of the calibrated Random Forest model against a perfectly calibrated reference line. **(b)** Calibration curve comparing the uncalibrated and calibrated Random Forest models.

The calibration curve in Figure 6a depicts the agreement between the actual and predicted probabilities of attrition for the Random Forest model after calibration. The blue curve denotes the observed probabilities against the calibrated model's predictions, while the orange dashed line represents a perfect calibration situation. If the curve lies close to the diagonal, it implies that the predicted probabilities correspond well to the actual ones. The calibrated model here is quite consistent with the ideal line, especially for the higher probability intervals; thus, the model produces fairly well-calibrated estimates. It allows HR professionals to understand predicted attrition more correctly, which leads to the execution of retention strategies in a more confident and precise way.

The calibration curve in Figure 6b illustrates the comparison between the employee attrition probability predictions from the Random Forest model before and after calibration. The uncalibrated model (blue line) moves away from the ideal diagonal, which means that the model's probability estimates do not agree with the actual attrition rates. After calibration (orange line), the curve was closer to the diagonal than before, showing that the probabilities were more accurate. So, the calibrated model offers more reliable risk estimates, which in turn is highly beneficial for HR managers to make retention decisions based on data. Accurate probabilities allow us to focus on the right employees, those who really are potential leavers.

**Figure 7:**
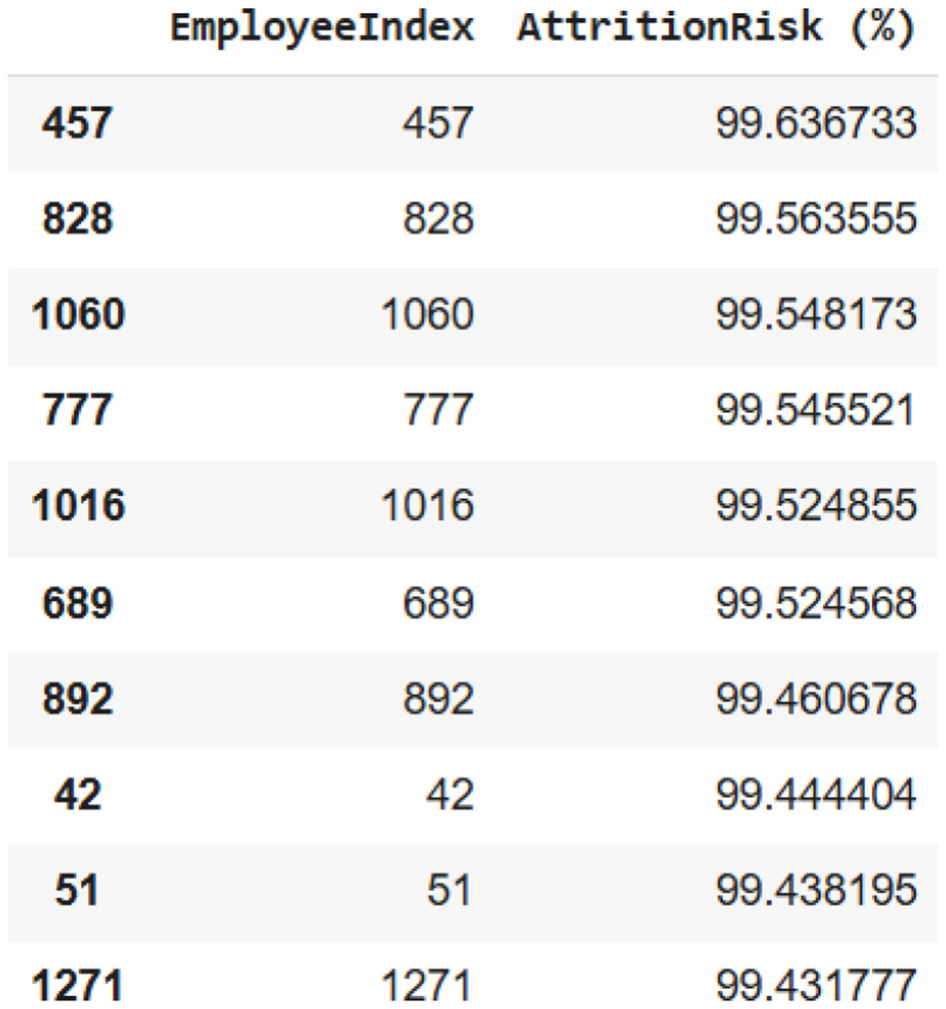
List of the top 10 at-risk employees identified after calibration.

**Figure 8:**
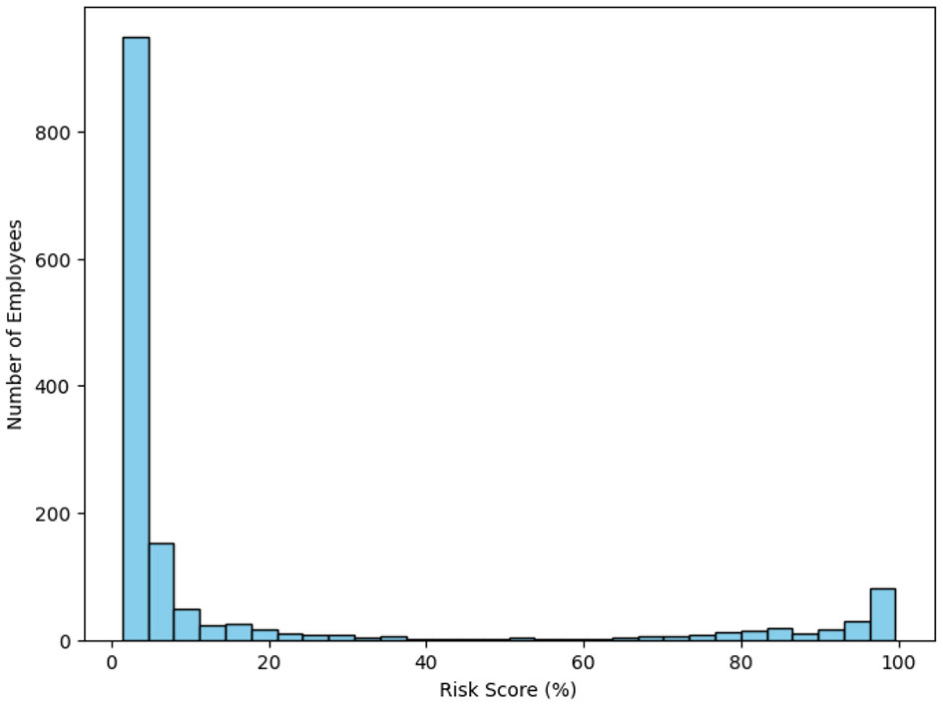
Distribution of calibrated attrition risk scores among employees.

Once the calibration was done, each employee was assigned a risk score. Figure 7 shows the top ten employees most likely to leave the company, thus making it possible for targeted retention initiatives. Figure 8 histogram indicates that most of the employees were characterized by low risk of attrition; however, a small number of employees had extremely high-risk scores, which, therefore, constitute the most critical segments for targeted HR intervention.

### 4.5 Explainable AI

**Figure 9:**
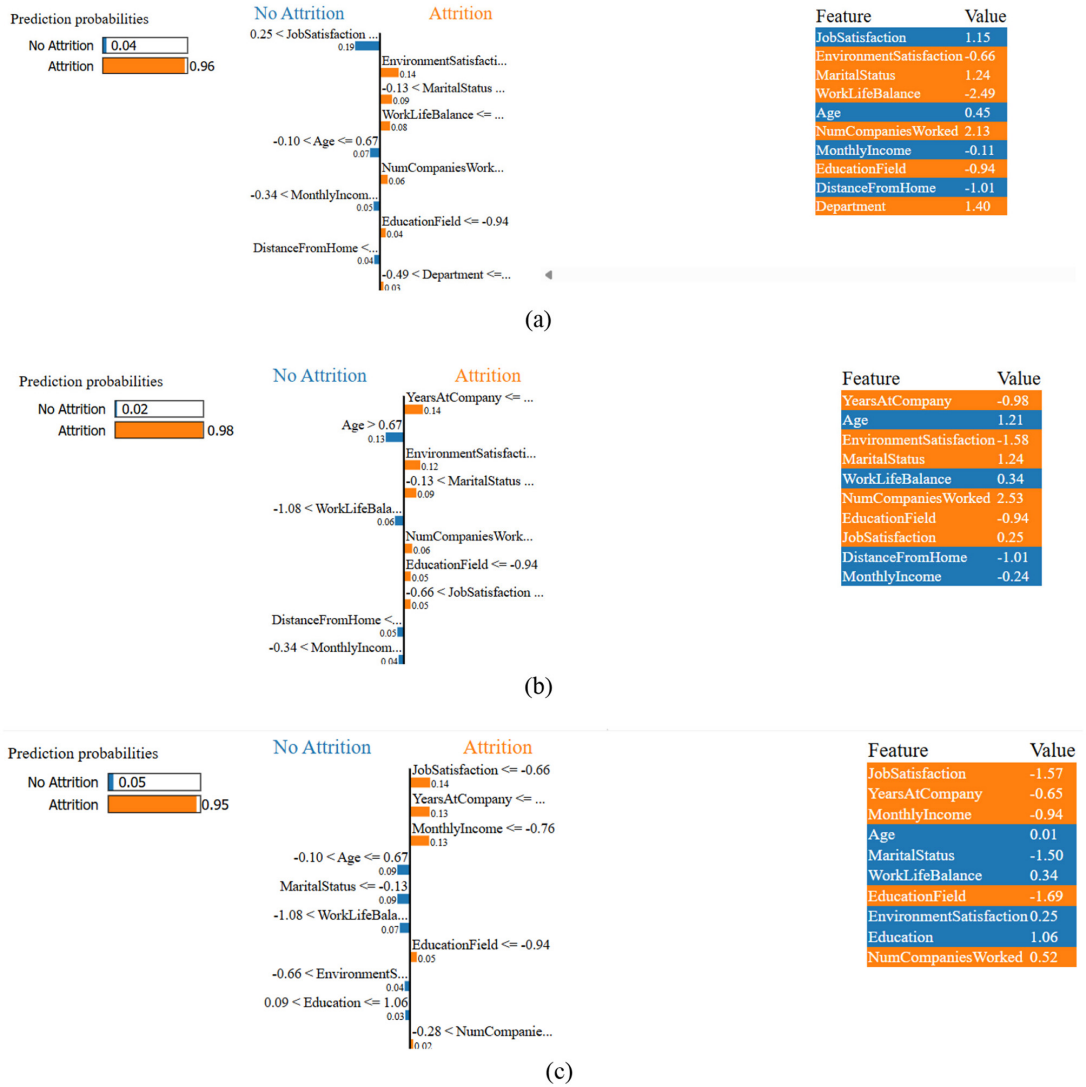
LIME-based explanations for individual employee attrition predictions from the test set are visualized at three distinct indices: **(a)** index 0, **(b)** index 50, and **(c)** index 100.

LIME was used to help close the gap between predictive modeling and actionable HR insights. For the individuals who are at high risk, LIME provides explanations in a way that is understandable by humans of the most important attrition factors, thus HR managers can develop personalized retention strategies. Figure 9 shows local explanations generated by LIME for employee attrition predictions from three representative test set indices. Each subplot indicates the top contributing features that influenced the model's prediction for a particular individual, thus providing interpretability in the decision-making process. In all three instances, the same set of features was found to be influential in predicting a high attrition risk. To be more precise, environmental satisfaction, marital status, education field, number of companies worked, years at the company, and job satisfaction were the factors that were most frequently pointed out as the key drivers. These trends imply that the model is able to capture significant and domain-relevant signals that are associated with employee turnover, thus strengthening its interpretability and potential use by the HR department in decision-making.

**Figure 10:**
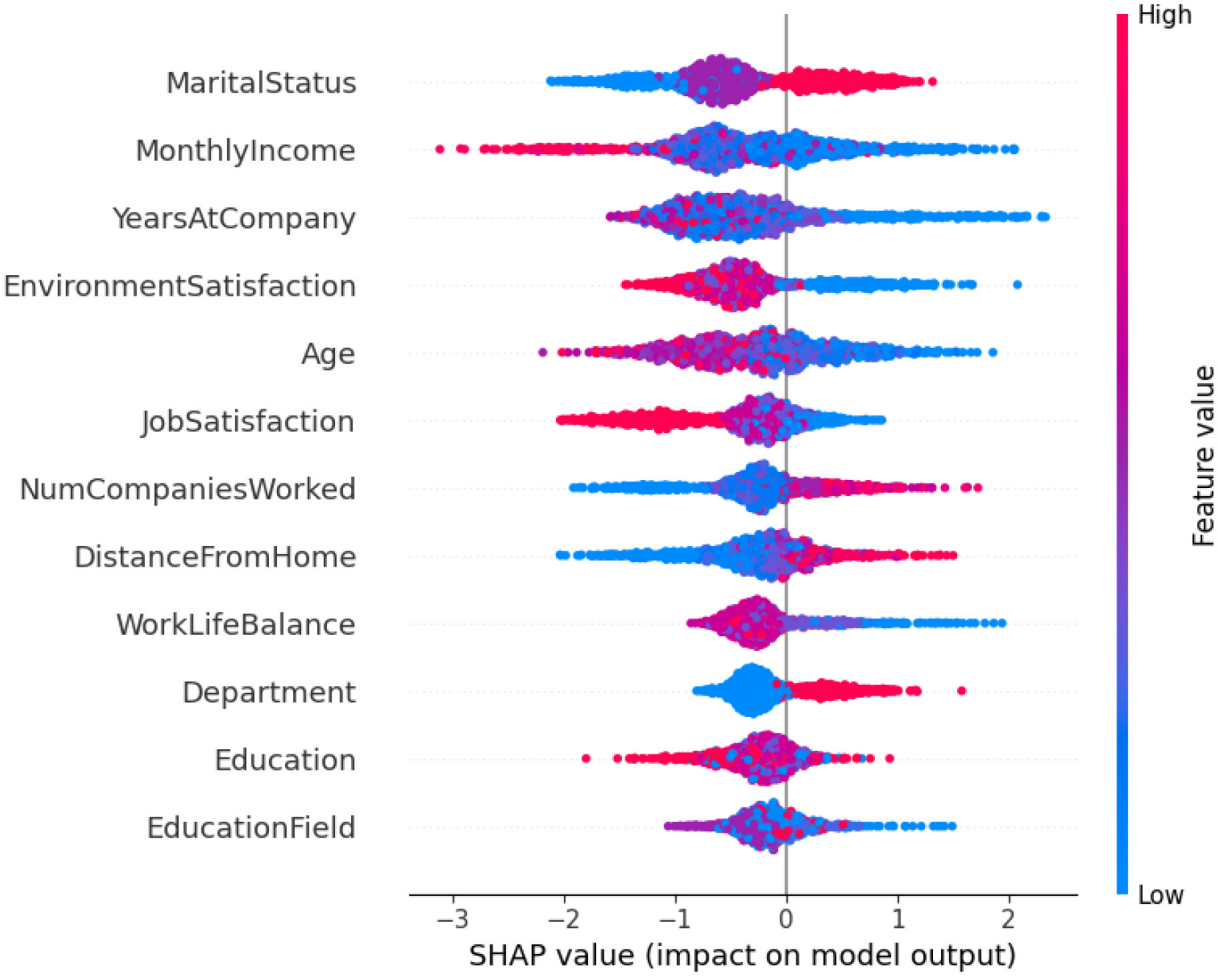
SHAP summary plot for Random Forest Model.

The SHAP summary plot in Figure 10 is a visual tool that helps us understand which features most influenced the model on a global scale and how each feature impacted the output of the Random Forest model for employee attrition. A single prediction's SHAP value is represented by each dot, showing how much a feature influenced the model's decision. For example, one of the most influential features was marital status, along with features such as monthly income, years at the company, environmental satisfaction, and job satisfaction, which showed a very strong influence, with both high and low values contributing differently to attrition risk. The feature value is indicated by the color gradient (blue to pink), thus revealing subtle relationships such as lower environmental satisfaction and fewer years at the company, tending to push predictions toward higher attrition. This plot supports the model's interpretability and also gives an insight into the main contributors that are consistent with domain knowledge.

**Figure 11:**
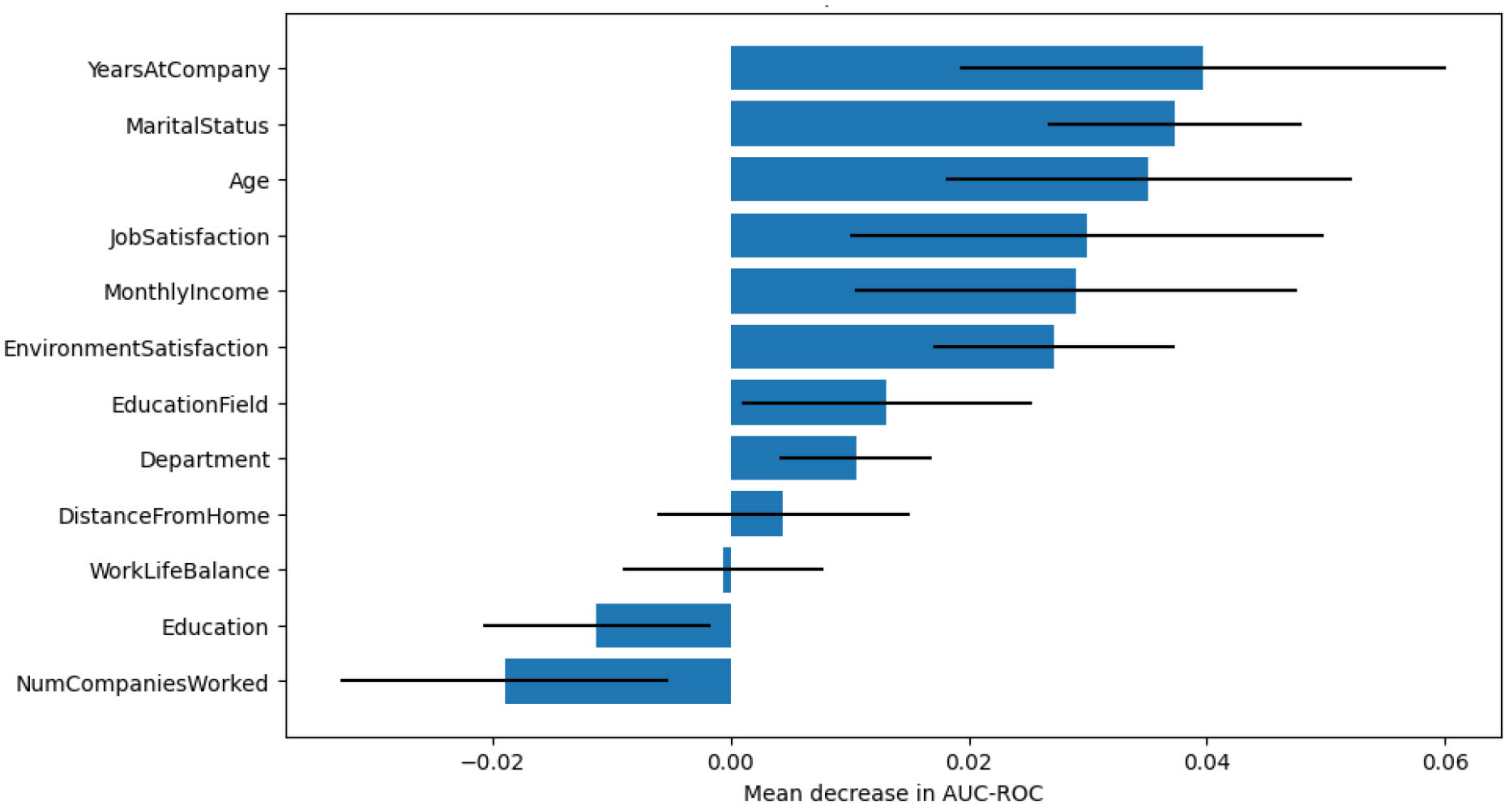
Permutation feature importance for Random Forest Model.

Figure 11 displays the permutation-based feature importance for the Random Forest classifier, which is a measure of the mean decrease in the ROC AUC after the features have been randomly shuffled. The horizontal bars show the relative influence of each feature on the performance of the model, while the error bars indicate the variation between the different shuffles. It is worth noting that the number of years with the company, marital status, age, job satisfaction, and monthly income were the top five features that most strongly predicted employee attrition. If these features were removed, the model would be less accurate; thus, these features played a very important role in the predictive outcomes. The findings are consistent with the theoretical framework, and the model being sensitive to tenure, personal circumstances, and workplace satisfaction is further confirmed.

In this study, random forest predictions are interpreted and explained using LIME, SHAP, and permutation feature importance. Additionally, these methods helped to identify the key drivers of employee attrition. Despite methodological differences, all three techniques consistently highlighted a core set of influential features: years at company, environment satisfaction, monthly income, and job satisfaction as primary contributors to attrition risk. LIME explained the model behavior in the local area, and it was an individual employee case; thus, the LIME results illustrated that combinations of low job satisfaction and low environmental satisfaction were the most frequent reasons behind the increase in risk scores of individual employees. SHAP explained the model behavior from both global and local points of view; it quantified the marginal impact of each feature, and thus it revealed that years at the company, environment satisfaction, and monthly income were the features that had the strongest overall influence for the whole dataset. Permutation feature importance can be considered as a confirmation of these conclusions because it is done by determining the decrease in the model's performance when these features are shuffled, which, therefore, indicates their predictive relevance. The agreement of these methods about the common features identified by them gives more trust to management that the model is stable, and it is a great tool for the implementation of retention strategies targeted at specific groups of employees.

### 4.6 Generalizability test

The proposed model was evaluated using the Bank Customer Attrition dataset, which contains demographic, transactional, and behavioral features representing customer activity and attrition status with 10,000 records. To assess the generalizability and robustness of the developed model, a Random Forest classifier with the same framework and optimal hyperparameters was trained. The model exhibited outstanding predictive power as it reached an accuracy of 99.85%, precision of 99.75%, recall of 99.51%, F1-score of 99.63%, and ROC-AUC score of 99.72%. Such uniformly high performance on all the evaluation metrics implies that the model has not only figured out the underlying data patterns very well but also has a strong generalization capability with new samples. Therefore, it consolidates the stability and robustness of the proposed framework for a real-world application.

### 4.7 Managerial implications

The introduced framework serves as a convenient, data-driven instrument for HR managers to strategically plan retention. It achieves this by fusing adjusted risk scores with understandable predictions, which in turn, facilitates the execution of interventions that are not only cost-effective but also morally correct. To illustrate, imagine a medium-sized IT firm struggling with the problem of losing employees who resign voluntarily at a high rate. HR, with the help of the model, can pinpoint the employees making up 10% of the workforce with the most significant risk of leaving. These employees may be given specially designed retention packages, such as the option to work from home, participation in a mentoring program, or a fast track for career development. Employees at moderate risk can receive the commitment program enrollment, while low-risk employees continue with the regular policies. This triage method enables HR to use resources in such a way that they have the most significant retention impact. One hypothetical policy simulation might consist of quarterly Attrition Risk Reviews, during which department heads receive dashboards depicting risk distributions and SHAP-based explanations for every employee who has been flagged. For instance, if the employee's dissatisfaction with the job and a long commute are the factors contributing to the highest risk, the managers could provide the option of remote work or change the employee's role. Gradually, HR will be able to monitor the results of the interventions and update the model accordingly to reflect changing workforce dynamics. By helping managers convert predictive insights into personalized plans, the framework is a tool for proactive workforce management. It gives the freedom to HR leaders to support their decisions with clear evidence, thus gaining trust from different levels of the organization and enhancing the employee experience.

### 4.8 Ethical considerations

Although predictive analytics is a set of robust tools that can be quite helpful in the decision-making of HR, it has issues that the consequences can be quite severe if not handled properly, like ethical problems. These issues concern the necessity of ensuring that the procedures are carried out in a fair, transparent, and accountable manner.

- Bias and Fairness. Machine learning models, which have been trained on past HR data, may unintentionally pick up biased patterns. For instance, they could link the age, gender, or marital status of a person with the likelihood of quitting a job. Such cases may result in discrimination and are dangerous if not adequately dealt with.
- Transparency and Explainability. One main problem of black-box models is that they do not reveal how they arrived at the result. In a sensitive area like HR, employees have the right to know the decisions made based on their careers. Using LIME and SHAP in this system makes the predictions understandable so that the HR department can provide the rationale for decisions and therefore be ethically defensible.
- Data Privacy. Information about employees is required to be treated with the utmost confidentiality. Companies should remove the identity from the most sensitive parts of data, only allow access to those who need it, and ensure they are abiding by the rules of data protection. Getting consent from and being transparent with employees about using their data are two things that ensure their trust.

This framework, through the incorporation of ethical provisions into the stages of modeling and deployment, is thus a way of encouraging the use of AI in an ethically responsible manner in human resources, that is, not violating human rights, and to be consistent with the moral principles of the organization while benefiting from the predictive power of the model.

### 4.9 Discussion

The research illustrates how the strategic value of attrition prediction systems can be markedly elevated by the HR integration of machine learning models that are calibrated, along with the use of explainable AI techniques. The Random Forest model, which was calibrated with the sigmoid method, not only achieved a high predictive accuracy but also delivered reliable probability estimates, as it was evident from the reduction in Brier Score. Contrary to the accuracy figures that most of the studies rely on, emphasis was made on the AUC-ROC score in this study so as to better measure the model's discriminative power at different risk thresholds. The certainty is, therefore, very important for HR managers who have to decide on retention efforts based on risk levels. The deployment of SHAP and LIME enabled the understanding of the model both at the global and local levels, thus giving the HR staff insights into the features that influence the highest attrition rates and the way they affect individual employees. To this end, openness enhances reliance on model outputs and thus supports decision-making that can be justified, particularly in the case of sensitive personnel matters. Furthermore, the framework is designed to overcome the identified greatest obstacles in the earlier literature, which include the absence of risk scores and a lack of employee-level explanations. By closing the gaps, the proposed system is not only about great technical performance, but also offers practical value for workforce planning and retention strategies.

## 5 Conclusion

This work develops a comprehensive, human-resource-focused machine learning framework to predict employee attrition. The model is less concerned with achieving the highest accuracy than with features such as calibrated probability estimation and explainable AI, which allow the framework to overcome the challenges of existing approaches that suffer from issues like lack of reliability, transparency, and usability of insights by managers.

The best overall model was determined from the set of classifiers to be the Random Forest, which, with a remarkable AUC-ROC score of 97.37%, was able to discriminate well between employees who would leave and those who would stay. In order to guarantee the dependability of the predicted probabilities, the sigmoid calibration method was employed, which lowered the Brier Score from 0.03873 to 0.03480, thus bringing the predicted probabilities closer to the actual attrition likelihoods. This step is especially important for HR managers who make decisions about the allocation of resources based on risk scores. A paired t-test was performed on stratified 5-fold cross-validation splits to statistically confirm the model choice. Random Forest was pitted against XGBoost. The test showed that the better performance of Random Forest was statistically significant and not due to chance.

The explainability of a model was improved with the use of LIME, SHAP, and permutation feature importance. These methods consistently pointed to company tenure, environmental satisfaction, income, and job satisfaction as the most influential variables in attrition prediction. LIME was used to explain the model locally at the level of a single employee, thus allowing personalized retention strategies to be devised. SHAP provided both a global and an individual perspective by measuring the incremental effect of each factor and thus facilitating strategic planning. Permutation feature importance confirmed these results by showing the decrease in the model's performance when the most important features were randomly shuffled.

From an organizational point of view, the model allows the HR department to single out the 10% of employees most likely to quit and thus to carry out targeted interventions, such as offering them flexible work arrangements, enrolling them in mentorship programs, or reviewing their compensation. Visual dashboards and SHAP-based explanations are additional tools that help managers make defensible, ethical decisions in a workforce characterized by the sensitivity of the issues.

The research moves beyond the technical challenge of model building toward practical HR implementation. This is achieved by implementing a high-performance predictive model that also scores risks in a calibrated manner and interprets them at different levels, thus giving HR the power to change from being attrition-driven to retention-planning-driven, and making these plans data-driven and proactive. The model provides an organization with a transparent, scalable, and ethically sound tool that aligns with their goals of maintaining a stable, engaged workforce.

### 5.1 Limitations and future scope of study

The proposed framework depicts a strong predictive performance; however, it is necessary to recognize certain limitations. The research relies on a small, synthetic dataset comprising 1,470 employee records, which might limit the model's capacity to generalize across different types of organizations. The insufficient sample size in this case heightens the possibility of overfitting, particularly if complex models such as Random Forest are utilized. Moreover, while the use of SMOTE for balancing classes is a good solution for the problem of minority class representation, it may also generate synthetic noise and change feature relations, thus increasing overfitting risks. The application of stratified cross-validation and calibration methods helps in reducing these issues; future work should still test the model on large and real-world datasets in their next work. Besides, the dataset is a snapshot, without changes over time, so the model cannot capture the employee behavior patterns that evolve. The framework also lacks the incorporation of behavioral or psychometric data, which could deepen prediction granularity and facilitate more tailored retention strategies.

Research can expand the framework by including multi-organizational and industry-specific datasets, thus making the model more generalizable. The use of temporal modeling techniques like survival analysis or recurrent neural networks will give the system an opportunity to identify attrition trends over time. Fairness improvement through bias identification and reduction methods will be very important as the concerned HR contexts are sensitive cases, like age, gender, or compensation. The deployment in real-time through interactive dashboards and the use of privacy-preserving methods such as federated learning will most likely enhance usability and scalability, thus allowing organizations to adopt it securely. Last but not least, the investigation of interpretable models or hybrid solutions may lead to finding a balance between ethical transparency and predictive power, which will increase managerial trust and practical impact.

## Data Availability

Publicly available datasets were analyzed in this study. This data can be found here: https://www.kaggle.com/datasets/yasserh/ibm-attrition-dataset.
